# Intra- and inter-observer reliabilities of cervical sagittal radiographic measurements among evaluators with different levels of training

**DOI:** 10.31744/einstein_journal/2025AO1947

**Published:** 2025-11-13

**Authors:** Guilherme Pianowski Pajanoti, Gustavo Henrique de Melo Ultchak, Jose Otavio Donadeli Tome, Matheus Neves Castanheira, Adham do Amaral Castro, Edgar Santiafo Valesin, Bruno Braga Roberto, Lucas Klarosk Ismael, Bruno Vieira Motter, Rodrigo Góes Medéa de Mendonça, Luciano Miller Reis Rodrigues, Matheus Pippa Defino

**Affiliations:** 1 Hospital Israelita Albert Einstein São Paulo SP Brazil Hospital Israelita Albert Einstein, São Paulo, SP, Brazil.; 2 Universidade de São Paulo Ribeirão Preto SP Brazil Universidade de São Paulo, Ribeirão Preto, SP, Brazil.

**Keywords:** Cervical vertebrae, Observer variation, Radiographic image interpretation, computer-assisted, Spine, Spinal fusion, Diskectomy, Students, medical, Surgeons, Radiologists, Reproducibility of results

## Abstract

**Objective:**

To assess the intra- and inter-observer reliabilities of cervical sagittal radiographic measurements performed by evaluators with varying levels of clinical experience.

**Methods:**

This study analyzed lateral cervical radiographs from 14 patients who had undergone single-level anterior cervical discectomy and fusion. Four evaluators, a spine surgeon, radiologist, spine surgery resident, and medical student, measured five sagittal alignment parameters (C2-C7 Cobb angle, T1 slope, cervical sagittal vertical axis, cervical sagittal vertical axis via C-shape, and T1- C2-C7 Cobb angle). Intra-observer variability was calculated as the deviation from the median measurements. Inter-observer reliability was assessed using intra-class correlation coefficients.

**Results:**

Intra-observer variability was lowest among experienced evaluators (13% for both the spine surgeon and radiologist) and highest for the medical student (19.4%). The intra-class correlation coefficients ranged from 0.54 to 0.96, indicating moderate to excellent agreement. When only the two most experienced evaluators were included in the analysis, intra-class correlation coefficients exceeded 0.88 for all parameters.

**Conclusion:**

Observer experience significantly influences the reliability of sagittal cervical radiographic measurements. Although less experienced evaluators demonstrate higher variability, their performance remains within acceptable limits. Standardized training protocols may help improve consistency across different levels of expertise.

## Highlights

Observer experience improves radiographic measurement reliability.

Spine surgeons and radiologists exhibited the highest intra-class correlation coefficients.

The cervical sagittal vertical axis showed the greatest inter-observer variability.

Standardized training may enhance measurement consistency.

## In Brief

This study evaluated the intra- and inter-observer reliabilities of cervical sagittal alignment measurements on lateral radiographs among observers with different experience levels. More experienced evaluators exhibited higher consistency, especially for complex parameters such as the cervical sagittal vertical axis.

## Graphical abstract

**Figure:**
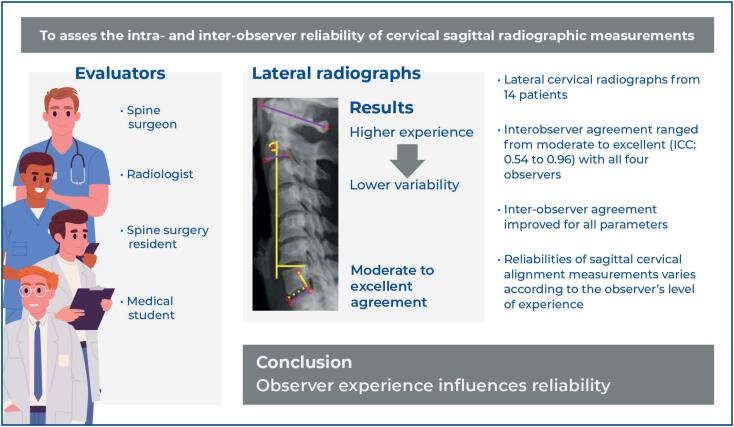

## INTRODUCTION

The sagittal alignment of the cervical spine is essential for an upright posture and horizontal gaze, as well as optimal energy expenditure, all of which contribute to quality of life.^(1)^ Alterations in cervical sagittal alignment and loss of lordosis have been linked to impaired function and worse patient-reported outcomes.^(2,3)^ Radiographic parameters commonly used to assess cervical alignment include the C2-C7 Cobb angle, C2-C7 cervical sagittal vertical axis (cSVA), and T1 slope. The clinical relevance of these values has been demonstrated through their correlation with postoperative and functional outcomes.^(3-7)^

The clinical applicability of these parameters depends on the reliability and reproducibility of the measurements, particularly in surgical planning and outcome assessments.^(7-9)^ However, multiple factors, including image quality, measurement technique, and, in particular, the level of experience of the observer, can affect measurement accuracy.^(10,11)^ Evaluating intra- and inter-observer variabilities is therefore essential to validate the consistency of radiographic measurements used in clinical decision making.

Although experienced observers are believed to provide more consistent assessments, few studies have specifically addressed the effect of training level on the reproducibility of sagittal cervical parameters.^(10-13)^ This gap in the literature highlights the need for a systematic analysis of observer-dependent variability, especially in academic and multidisciplinary clinical settings.

This study aimed to evaluate the intra- and inter-observer reliabilities of key cervical sagittal parameters measured by evaluators with different levels of experience. Lateral cervical radiographs of patients who underwent single-level anterior cervical discectomy and fusion (ACDF) using standalone cages were selected for analysis. This manuscript should provide information on the consistency of radiographic measurements among observers with distinct training backgrounds.

## OBJECTIVE

To assess the intra- and inter-observer reliabilities of cervical sagittal radiographic measurements performed by evaluators with varying levels of experience.

## METHODS

This study was reviewed and approved by the Research Ethics Committee of *Hospital Israelita Albert Einstein* (CAAE: 78861624.1.0000.0071; # 7.639.625). The requirement for informed consent was waived because the study involved only anonymized radiographic images.

The medical records of patients who underwent single-level ACDF at *Hospital Israelita Albert Einstein* in 2023 and 2024 were retrospectively reviewed. Patients aged ≥18 years and with available adequate-quality lateral cervical spine radiographs were included in the study. Patients with incomplete images or a history of cervical spinal surgery were excluded from the study. A total of 14 patients met the eligibility criteria and were included in the analysis.

Radiographs were acquired using a standardized imaging protocol to ensure consistency in the lateral cervical spine projection. All clinical and imaging data were anonymized by an independent professional who was not involved in the study; the evaluators were blinded to the identities of the patients and timing of the images. Anonymized images were distributed to the four evaluators for assessment using Surgimap software (Globus Medical, Methuen, MA, USA).

The evaluators consisted of two experienced professionals, a spine surgeon and a radiologist, and two less experienced individuals, a spine surgery resident and a medical student. Each evaluator was instructed to identify specific anatomical landmarks according to predefined guidelines.

**Figure 1:**
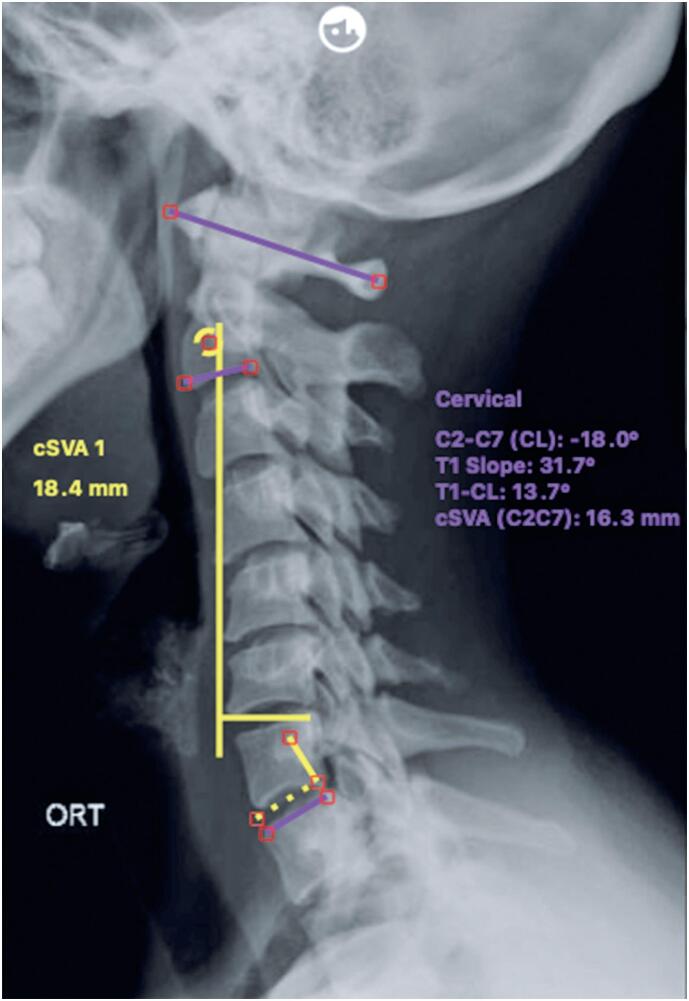
Cervical sagittal alignment parameter measurements as performed on radiographs using Surgimap software

The following parameters were evaluated (12, 13): C2-C7 Cobb angle, a measure of cervical lordosis (CL); T1 slope; cSVA; cSVA (C2-C7); and T1-C2-C7 Cobb angle (T1-CL). The C2-C7 Cobb angle is the angle formed between a line parallel to the inferior endplate of C2 and a line parallel to the superior or inferior endplate of C7. The T1 slope represents the angle between the superior endplate of T1 and the horizontal plane. The cSVA is defined as the shortest distance from a vertical plumb line drawn from the center of the C2 vertebral body to the posterosuperior corner of C7, as measured directly by the evaluator. The cSVA (C2-C7) is the cSVA as measured indirectly using the "C-shape" software tool. The T1-CL is calculated by subtracting the CL angle (C2-C7 Cobb) from the T1 slope. The sagittal cervical parameter measurements are illustrated in figure 1.

cSVA: cervical sagittal vertical axis.

This study focused exclusively on evaluating intra- and inter-observer reliabilities. Therefore, comparisons between pre- and post-operative images were not performed, although these data were available.

Intra-observer variability was quantified as the absolute deviation of the measurement of each observer from the median value of the parameter.

Inter-observer reliability was assessed by calculating intra-class correlation coefficients (ICCs). Intra-class correlation coefficients of <0.50, 0.50-0.75, 0.75-0.90, and >0.90 are considered to indicate poor, moderate, good, and excellent reliabilities, respectively.^(12,13)^

## RESULTS

### Intra-observer agreement

The average intra-observer variability was 13.4% (mean absolute error [MAE]=1.9) for the spine surgeon, 13.1% (MAE=1.78) for the radiologist, 15.2% (MAE=1.88) for the spine resident, and 19.4% (MAE=2.66) for the medical student. The proportions of measurements with less than 20% deviation from the average value of the parameter were 69.2%, 73.5%, 73.1%, and 61.3%, respectively. The detailed percentage errors for each parameter and observer are listed in table 1.

**Table 1:** Intra-observer variability in cervical spine parameter measurement

Observer	Parameter	Mean percentage error (%)
Spine surgeon	C2-C7 (CL)	9.4
Radiologist	C2-C7 (CL)	11.7
Spine resident	C2-C7 (CL)	12.8
Medical student	C2-C7 (CL)	12.7
Spine surgeon	cSVA	17.9
Radiologist	cSVA	18.5
Spine resident	cSVA	16.4
Medical student	cSVA	38.9
Spine surgeon	cSVA (C2-C7)	26.0
Radiologist	cSVA (C2-C7)	19.1
Spine resident	cSVA (C2-C7)	21.2
Medical student	cSVA (C2-C7)	29.5
Spine surgeon	T1 slope	4.0
Radiologist	T1 slope	4.7
Spine resident	T1 slope	5.6
Medical student	T1 slope	5.0
Spine surgeon	T1-CL	9.8
Radiologist	T1-CL	11.7
Spine resident	T1-CL	20.4
Medical student	T1-CL	10.4

Data are expressed as the mean percentage error in relation to the median of each observer.

cSVA: cervical sagittal vertical axis.

### Inter-observer agreement

The ICCs are presented in table 2. The T1 slope (ICC=0.937) and C2-C7 Cobb angle (ICC=0.963) demonstrated excellent reliability, with the T1-CL (ICC=0.895) showing good reliability. In contrast, the cSVA (ICC=0.549) and cSVA (C2-C7) (ICC=0.544) showed only moderate reliability. When only the two most experienced observers (the spine surgeon and radiologist) were included in the analyses, ICCs increased across all parameters (Table 3), with the cSVA reaching 0.974 and T1-CL achieving 0.949, indicating excellent agreement.

**Table 2:** Intra-class correlation coefficients for cervical spine parameter measurements performed by four evaluators

Parameter	ICC	95%CI	p value	Reliability
T1 slope	0.937	0.87–0.98	1.94e-22	Excellent
C2–C7 (CL)	0.963	0.92–0.99	1.08e-26	Excellent
cSVA	0.549	0.12–0.83	2.60e-15	Moderated
cSVA (C2–C7)	0.544	0.12–0.82	2.14e-15	Moderated
T1-CL	0.895	0.79–0.96	1.99e-17	Excellent

95%CI: 95% confidence interval; cSVA: cervical sagittal vertical axis; ICC: intra-class correlation coefficient.

**Table 3:** Intra-class correlation coefficients for cervical spine parameter measurements performed by the two most experienced evaluators

Parameter	ICC	95%CI	p value	Reliability
T1 slope	0.956	0.87–0.99	1.48e-08	Excellent
C2–C7 (CL)	0.964	0.9–0.99	4.50e-09	Excellent
cSVA	0.974	0.92–0.99	5.66e-10	Excellent
cSVA (C2–C7)	0.883	0.66–0.99	2.40e-06	Good
T1-CL	0.949	0.85–0.99	4.85e-08	Excellent

95%CI: 95% confidence interval; cSVA: cervical sagittal vertical axis; ICC: intra-class correlation coefficient.

## DISCUSSION

This study demonstrated that the reliability of sagittal cervical alignment measurements increases along with the level of experience of the evaluator. Inter-observer agreement ranged from moderate to excellent for all four evaluators to good or excellent for the two senior evaluators.

Direct and indirect cSVA measurements showed the greatest variability. These parameters reached only moderate levels of inter-observer reliability when all four evaluators were analyzed. These findings align with those of previous reports, which suggested that the cSVA is particularly sensitive to variation due to the difficulty in consistently identifying precise anatomical landmarks, in particular the center of the C2 vertebral body, on lateral radiographs.^(14-16)^ Variations in image quality and subtle differences in head positioning can further increase this difficulty.

As expected, the average measurement error increased as the level of observer experience decreased. However, the overall intra-observer agreement remained within acceptable ranges across all experience levels, suggesting that even less experienced evaluators, when properly guided, can produce reasonably consistent measurements. These findings support the implementation of standardized measurement protocols and structured training, especially in academic and clinical environments, where junior professionals are actively involved in image analysis.^(17)^

Few studies have specifically investigated the effect of training level on the reproducibility of cervical sagittal parameters. This study contributes novel evidence by showing that although automated tools like Surgimap can aid in measurement standardization, the expertise of the evaluator remains a crucial determinant of reliability. Accurate radiographic interpretation therefore depends not only on the tools used but also on the training and experience of the evaluator.

Inaccurate measurements can lead to erroneous clinical interpretations, suboptimal surgical planning, or flawed research conclusions. Accurate and reproducible radiographic measurements are essential for preoperative planning, postoperative evaluation, and the long-term monitoring of cervical alignment, particularly in patients undergoing procedures such as ACDF. Thus, proper training is not merely an academic concern; it is directly linked to patient care quality.

One limitation of this study was its small sample size, which may have reduced the statistical power of the analysis. Furthermore, although lateral radiographs are widely used for analysis of cervical sagittal parameters in clinical practice owing to their accessibility and low cost, they present inherent limitations for spatial measurements, particularly distance-based parameters such as the cSVA. Future studies using more advanced imaging technologies such as computed tomography or EOS imaging and including a larger number of evaluators across broader training levels may provide deeper insights and improve external validity.

## CONCLUSION

The findings of this study demonstrate that the reliability of cervical sagittal parameter measurements, particularly those of the cervical sagittal vertical axis, varied according to the level of experience of the evaluator. These findings highlight the importance of training and standardization in radiographic assessments. Given the critical role of accurate imaging in surgical planning and postoperative evaluation, the appropriate training of professionals involved in image interpretation is essential to ensure consistency and optimize patient outcomes. Future studies involving larger sample sizes and advanced imaging technologies may further enhance measurement accuracy and support the development of more robust, standardized protocols for evaluating cervical sagittal alignment.
